# Mitochondrial dysfunction is a key link involved in the pathogenesis of sick sinus syndrome: a review

**DOI:** 10.3389/fcvm.2024.1488207

**Published:** 2024-10-29

**Authors:** Xinxin Shi, Liming He, Yucheng Wang, Yue Wu, Dongming Lin, Chao Chen, Ming Yang, Shuwei Huang

**Affiliations:** ^1^ Department of Cardiology, The First Affiliated Hospital of Zhejiang Chinese Medical University (Zhejiang Provincial Hospital of Chinese Medicine), Hangzhou, China; ^2^ The First Clinical Medical College, Zhejiang Chinese Medical University, Hangzhou, China; ^3^ Department of Cardiology, Hangzhou TCM Hospital of Zhejiang Chinese Medical University, Hangzhou Hospital of Traditional Chinese Medicine, Hangzhou, China; ^4^ Department of Cardiology, The Second Affiliated Hospital of Zhejiang Chinese Medical University, Hangzhou, China

**Keywords:** Ca^2+^ clock, fibrosis, ion channel protein, membrane clock, mitochondria, review, sick sinus syndrome

## Abstract

Sick sinus syndrome (SSS) is a grave medical condition that can precipitate sudden death. The pathogenesis of SSS remains incompletely understood. Existing research postulates that the fundamental mechanism involves increased fibrosis of the sinoatrial node and its surrounding tissues, as well as disturbances in the coupled-clock system, comprising the membrane clock and the Ca^2+^ clock. Mitochondrial dysfunction exacerbates regional tissue fibrosis and disrupts the functioning of both the membrane and calcium clocks. This plays a crucial role in the underlying pathophysiology of SSS, including mitochondrial energy metabolism disorders, mitochondrial oxidative stress damage, calcium overload, and mitochondrial quality control disorders. Elucidating the mitochondrial mechanisms involved in the pathophysiology of SSS and further investigating the disease's mechanisms is of great significance.

## 1 Introduction

Sick sinus syndrome (SSS) encompasses a group of syndromes characterized by impaired pacing function and/or conduction of electrical impulses due to dysfunction of the sinoatrial node and its surrounding tissue (1, 2). The disease tends to occur in the elderly (3), with an overall annual incidence of nearly 1 per 1,000 individuals aged 45 years and above (4), and approximately 1 per 600 individuals aged 65 years and above (5). It is reported that the incidence of SSS is potentially associated with age and race (6). The onset of SSS is typically insidious and progresses slowly, often rendering early diagnosis challenging, as mild cases may remain asymptomatic. However, severe cases can manifest as sinus arrest, Adams-Stokes syndrome, or sudden death (7, 8). SSS is also one of the main indications for permanent pacemaker implantation (9, 10), but recent studies indicate that while pacemaker implantation ameliorates symptoms, it does not significantly enhance survival rates (11), and there are several main disadvantages such as high cost, unsatisfactory treatment effect and great intraoperative risk (12). The pathogenesis of SSS remains incompletely understood, so it is significant to delve into the core link of the disease mechanism. Mitochondrial dysfunction is implicated in the pathogenesis of various arrhythmia diseases (13), while the attention of SSS in arrhythmia-related diseases is far less than that of atrial fibrillation. There is a lack of comprehensive review and summary. This paper examines the pivotal role of mitochondrial dysfunction in the pathogenesis of SSS.

## 2 Basic mechanisms of SSS

### 2.1 Fibrosis of the sinoatrial node and its surrounding area

The cells in the sinoatrial node are composed of pacemaker cells (P cells), transition cells (T cells), cardiac fibroblast (CF), and atrial myocytes. There are 35%–55% fibrotic tissues in this region (14), and the necessary degree of fibrosis in the sinoatrial node tissues plays a role in maintaining the structural integrity and electrical insulation in the conduction process. Such structural characteristics ensure that the electrical signal is effectively transmitted to the myocardium via the sinoatrial conduction pathway (SACP) (15–17).

In general, the extent of degenerative fibrosis of the sinoatrial node exhibits a positive correlation with advancing age. As age increases, there is a notable decrease in sinoatrial node cells and/or an increase in fatty tissue infiltration (14, 18, 19), disrupting the continuity between the sinoatrial node and the surrounding atrial myocardium (20), which is considered the most significant intrinsic cause of SSS (21).As well as the atrial muscle tissue around the sinoatrial node, it's fibrotic process resulting in slowed conduction and atrial systolic dysfunction, which concomitantly increases the risk of atrial fibrillation (22). The two genetically engineered mice, ROSA-eGFP-DTA and HCN4-KiT Cre, cultured by Stefan Herrmann et al., accurately reflected the histopathological results of human with SSS, wherein tissue degenerative fibrosis in the sinoatrial node resulted in abnormal cardiac pacing (23).Pathological fibrosis of the sinoatrial node is also one of the etiologies of SSS (15, 24), CF within the sinoatrial node secrete substantial amounts of extracellular matrix (ECM) in response to angiotensin II (AngII), inflammatory injury, oxidative stress, overload, and an acidic environment, culminating in pathological fibrosis of the sinoatrial node (25–27). The TGF-β/SMAD signaling pathway, modulated by AngII, is a well-established mediator of interstitial fibrosis (28–30). The expression of TGF-β1 related genes is implicated in the pathogenesis of SSS (31), with elevated levels of TGF-β1 and Smad2 proteins observed in atrial muscle remodeling associated with SSS (32). The SSS mouse model developed by Chen et al. with a micro-osmotic pump to continuously administer AngII, further simulates tissue pathological fibrosis in the sinoatrial node stimulated by AngII (33).

### 2.2 Dysfunction of the coupled-clock system

Lakatta's laboratory proposed the coupled-clock system, comprising the integration of the “Ca^2+^ clock” and the “membrane clock”, which is regarded as a crucial mechanism for the functional operation of the sinoatrial node pacemaker (34, 35). The strict synchronization between the two clocks ensures that the sinoatrial node beats steadily and rhythmically, and the “membrane clock” is the periodic activity of the major ion currents on the cell membrane (Table 1), including the delayed rectifier potassium current (I_k_), funny current (I_f_), L-type voltage-gated calcium channel current (I_Ca,L_), T-type voltage-gated calcium channel currents (I_Ca,T_), and so forth (51). Among them, I_f_ controlled by hyperpolarization-activated cyclic-nucleotide gated (HCN) channels plays a dominant role (41, 52). The “Ca^2+^ clock” primarily involves the inward current generated by the sodium-calcium exchanger (NCX), which maintains intracellular Ca^2+^ concentrations during diastole. Ca^2+^ serves as a mediating signal between the membrane clock and the Ca^2+^ clock, facilitating current conduction within sinoatrial node cells (53). Changes in the initial membrane potential lead to the opening of L-type Ca^2+^ channels, and Ca^2+^ influx elevates intracellular Ca^2+^ concentration. This intracellular Ca^2+^ can be recycled from the cytoplasm into the sarcoplasmic reticulum (SR) via the sarcoplasmic reticulum Ca^2+^-ATPase (SERCA), which activates SR ryanodine receptors (RyRs) to release Ca^2+^, thereby generating local Ca^2+^ release (LCR). The LCR subsequently activates the NCX, and the resultant internal Sodium-calcium exchanger current (I_NCX_) generates activates I_Ca,L_ again, and a new action potential is formed (54, 55). Any impairment within the coupling of the membrane clock and Ca^2+^ clock will disrupt the pacing currents of the sinoatrial node, adversely affecting the regular and stable rhythmicity of sinoatrial node contractions, thereby precipitating the occurrence of SSS (41, 56–59).

**Table 1:** Major ion currents and their regulatory proteins on the membrane clock.

Participating in the process of cardiac electrical activity and current direction	Ion current	Ion channel protein	Reference
Participating in depolarization, total electrical flow inward	I_f_	HCN1, HCN2, HCN3, HCN4 (mainly HCN4, HCN3 low expression）	(36, 37)
	I_Ca,L_	CaV1.2, CaV1.3	(38–40)
	I_Ca,T_	CaV3.1, CaV3.2, CaV3.3 (mainly CaV3.1, CaV3.3 is rarely expressed)	(41–43)
	I_NCX_	NCX	(44)
	I_Na_	Nav1.5	(45)
Participating in repolarization, total electrical flow outward	I_Kto_	Kv4.2, Kv4.3, Kv1.4	(46)
	I_Kr_	hERG	(47)
	I_Ks_	KvLQT1	(48)
	I_KAch_	Kir2.1, Kir2.2, Kir2.3, Kir2.4 (Kir2.3 low expression)	(41)
	I_K1_	Kir3.1, Kir3.4	(49)
	I_KATP_	Kir6.2	(50)

In summary, the coupling mechanism of the coupled-clock system is essential for the sustained conduction activity of the sinoatrial node. Adequate levels of tissue fibrosis are crucial for maintaining the insulating properties of cardiac electrical conduction, while the interplay between conduction and insulation is vital for the normal functioning of the sinoatrial node. Remodeling of the sinoatrial node often results in fibrosis of both the sinoatrial node and the surrounding atrial muscle tissue (15, 60–65), induced by conditions such as atrial fibrillation, atrial flutter, heart failure, myocardial infarction. A small group of patients with SSS caused by family genetic factors are associated with regional tissue fibrosis, such as TGF-β1T869C gene (31), while the majority of cases are linked to mutations in ion channel-related genes, with SCN5A and HCN4 identified as two prominent pathogenic genes associated with SSS (66–72), and others include KCNG2 (73), SCN10A (74), GNB2 (75, 76). As well as endogenous metabolites like adenosine (77, 78)and adrenaline (79–81), are closely associated with sinoatrial node fibrosis and dysfunction of the coupled-clock system.

## 3 Mitochondria and mechanisms of SSS

### 3.1 Mitochondrial energy metabolism and mechanisms of SSS

Mitochondria, often known as the energy factories of cells, are responsible for the production of adenosine triphosphate (ATP) through oxidative phosphorylation, thereby supplying energy to the cell. In the context of cardiac physiology, sinoatrial node cells, functioning as the heart's natural pacemaker, must continuously generate electrical impulses, resulting in a heightened demand for energy. The mechanism of SSS and mitochondrial energy metabolism are reflected in the following aspects (Table 2).

**Table 2:** Summary of mitochondrial energy metabolism and mechanisms of SSS.

Action target		Mechanism of action
ATP-related ion channels	K_ATP_ channel	Involve in action potential formation, and influence I_Ca,L_
	VRAC	Mediate chloride ion currents (I_Cl, swell_), and involve in regional tissue fibrosis
ATP-related ion pumps	SERCA	Maintain Ca^2+^ homeostasis within the cytoplasm and organelles, and involve in LCR formation
	NKA	Resulting Na + concentration difference acts as the driving force of NCX, forms I_NCX_
cAMP derived from ATP	HCN4, L-type Ca^2+^ channel	Trigger rhythmic action potential
	PKA/CaMKII	Drive LCR
	cAMP/PKA, cAMP/PGE1, TGFβ/Smad	Regulate the fibrosis process

#### 3.1.1 ATP-related ion channels

##### 3.1.1.1 ATP-sensitive potassium channel (K_ATP_ channel)

K_ATP_ channel comprises Kir6 main subunit and SuR auxiliary subunit (82), and participates in potassium ion current in sinoatrial node cells (83). Its activity is modulated by intracellular ATP concentrations; elevated ATP levels inhibit the channel, preventing its involvement in action potential formation and excitation-contraction coupling. Conversely, a decrease in ATP concentration leads to channel activation, facilitating K^+^ efflux, which accelerates repolarization and shortens the action potential plateau (84–86). In addition to K_ATP_ channels distributed on the cell membrane, which participate in the formation of membrane clock currents, K_ATP_ channels distributed on the inner mitochondrial membrane are closely related to mitochondrial energy metabolism, mitochondrial membrane potential maintenance, apoptosis inhibition, and Ca^2+^ overload alleviation, thereby sustaining the homeostatic state of the intracellular environment (87–89). The activity of K_ATP_ channel is regulated by mitochondrial energy metabolism and synchronously participates in mitochondrial energy metabolism and Ca^2+^ homeostasis, thereby influencing I_Ca,L_ (90), and repeatedly affecting the coupled-clock system mechanism which mediates the pacemaker currents of sinoatrial node cells.

##### 3.1.1.2 Volume-regulated anion channel (VRAC)

VRAC is expressed in atrial myocytes, ventricular myocytes and P cells, and is involved in cardiac physiology and pathophysiological processes (91, 92). VRAC activation occurs in response to cellular swelling, hence it is also referred to as the swelling-activated chloride channel. The primary component of VRAC is leucine-rich repeat protein 8 (LRRC8)A, commonly known as Swell1 (93), is essential for regulating cellular volume reduction, maintaining cell volume homeostasis, and mediating chloride ion currents (I_Cl, swell_) (94). Activation of VRAC requires the participation of ATP (95, 96), and the function is reversibly inhibited in hypoxic environment and under the action of mitochondrial inhibitors (97). Interestingly, VRAC is regulated by mitochondrial energy metabolism; inhibition of VRAC consequently affects the mitochondrial electron transport chain, thereby impairing ATP production (98). On the other hand, normally functioning VRAC channels also play an important role in CF (99). Given their role as energy-demanding ion channel proteins, it is plausible to suggest that metabolic disorders arising from mitochondrial dysfunction may provoke inflammatory responses in the sinoatrial node or contribute to increased extracellular matrix deposition, thereby exacerbating regional tissue fibrosis.

#### 3.1.2 ATP-related ion pumps

##### 3.1.2.1 SERCA

SERCA is a crucial ion pump integral to the Ca^2+^ clock mechanism of the sinoatrial node. Its primary function is to restore intracellular Ca^2+^ levels and maintain calcium homeostasis within the cytoplasm and organelles, such as mitochondria and the SR. The stable release of Ca^2+^ in the SR ensures the stable LCR of the Ca^2+^ clock. SERCA has a high affinity with ATP (100), while ATP consumption is accompanied by Ca^2+^ recycling (101, 102), which can negatively regulate SERCA activity by restricting ATP produced in mitochondria (103). Meanwhile, Claudia Rodriguez et al. also demonstrated that AMP-activated protein kinase (AMPK), which is involved in mitochondrial protection, can stimulate the ATP-generating pathway and restore homeostasis to activate SERCA activity (104). This indicates that mitochondria play a significant role in cytoplasmic Ca^2+^ recovery through SERCA based on energy metabolism, and further participate in the coupled-clock system's coupling mechanism. The ability of sinoatrial node cells to generate larger and rhythmic LCRs should be linked with increased abundance of SERCA (55), while the expression level and activity of SERCA tend to decline with age (105), which also proves that mitochondria-driven attenuation of SERCA during aging is one of the mechanisms of SSS.

##### 3.1.2.2 Na^+^/K^+^-ATPase (NKA)

NKA is an ion pump that consumes ATP to maintain the balance of sodium (Na^+^) and potassium (K^+^) ions within the cell (106). It works in concert with other ion channels and pumps to regulate calcium (Ca^2+^) homeostasis within the SR, which is crucial for both SR Ca^2+^ equilibrium and membrane depolarization in sinoatrial node cells. This regulation ensures the maintenance of the resting membrane potential and the rhythmic function of the sinoatrial node (107, 108). When the concentration of cytoplasmic Ca^2+^ is low, SERCA is in a low activity state, while NKA, which is in a high activity state, can account for 30% of all ATPases (109), and participate in the transport of Na^+^ and K^+^. The resulting Na^+^ concentration difference acts as the driving force of NCX (110–112). The whole process is regulated by intracellular Ca^2+^ signaling (111), and eventually I_NCX_ is formed (113), which is vital to the electrical function of the heart (114). Gvantsa Chkadua et al. noted that NKA activity is regulated not only by mitochondrial ATP but also by cytochrome c (Cyt C) released from mitochondria, which mediates non-apoptotic effects. NKA is activated by low Cyt C concentrations and inhibited at higher concentrations (115). Norbert A. Dencher et al. observed impaired mitochondrial function and significantly reduced NKA expression in aged rats or those with age-related diseases (116). Therefore, NKA is intricately linked with mitochondrial function and is a potential target for mitochondrial-mediated cardiac protection (117).

#### 3.1.3 Cyclic adenosine monophosphate (cAMP) derived from ATP is involved in the cardiac electrical activity of sinoatrial node cells

cAMP, mainly derived from ATP, is formed by the cyclization reaction of ATP after the removal of one pyrophosphate (two phosphorus atoms) under the catalysis of adenylyl cyclase (AC) (118). It is widely present in cells and plays a crucial role in various physiological processes, notably acting as an intracellular “second messenger” in the sinoatrial node (81, 119). cAMP/protein kinase A (PKA) signal is an important mechanism driving the coupled-clock system of sinoatrial node cell membrane to trigger rhythmic action potential (120), and the efficacy of this mechanism diminishes with age due to a reduction in cAMP levels (80). Therefore, many iron channels on the sinoatrial node cell membrane are regulated by cAMP, with the most dependent being HCN4 (121–124), L-type Ca^2+^ channels (125–127). Regarding the regulation of the Ca^2+^ clock, the occurrence of LCR is dependent on the phosphorylation of cAMP downstream PKA and Ca^2+^/calmodulin-dependent protein kinase II (CaMKII) (127–129), and cAMP also plays a regulatory role in inhibiting tissue fibrosis (130). Age-related cardiac contraction decline is partly attributed to the desensitization of β-adrenergic/cAMP signaling. Enhancing cardiac cAMP bioavailability and PKA activity has been shown to improve contractile function in mice, potentially alleviating fibrosis and cardiac tissue remodeling (131). Besides that, cAMP-elevating receptor agonist prostaglandin E1 (PGE1) can inhibit cardiac fibroblast proliferation (132). Alternatively, cAMP inhibits the downstream TGFβ/Smad signaling pathway, reduces the expression of α-SMA, alleviates ECM deposition, thus regulating the fibrosis process (133–137).

In addition, although the cystic fibrosis transmembrane conductance regulator (CFTR) has not been reported to be related to the SSS mechanism, it holds potential as a target protein for future investigations into its role in the electrophysiological processes and fibrosis progression within the sinoatrial node and adjacent tissues. CFTR is extensively expressed in the heart (138), which requires ATP hydrolysis for energy (139–141), and is also shown as cAMP-dependent (142), and accompanied by current signals I_Cl,cAMP_ during chloride ion transport (143, 144). What is more interesting is that gene mutations resulting in the mistranslation of CFTR can directly cause cystic fibrosis (139), suggesting a subtle connection between the fundamental mechanisms of SSS and cystic fibrosis, whether by coincidence or inevitability.

### 3.2 Mitochondrial oxidative stress and SSS

Aging and a variety of cardiovascular diseases are believed to be related to mitochondrial oxidative stress. For instance, mice with a germline deletion of the Ndufs4 subunit of respiratory complex I exhibit chronic arrhythmias, atrioventricular block, and other sinoatrial node dysfunctions. Targeted anti-oxidative stress therapy has been shown to ameliorate chronic arrhythmias and prolong their lifespan (145). The role of mitochondrial oxidative stress in the pathogenesis of SSS may be related to the following aspects (Table 3).

**Table 3:** Summary of mitochondrial oxidative stress and SSS.

Action target	Mechanism of action
Membrane clock ion channel proteins	Inhibit the function of membrane clock ion channel proteins (HCN4, KATP, SCN5A, KCNA5), and influence ion currents, but if the antioxidant treatments will ease
CaMKII	Affect Ca^2+^ clocks LCR process, and even further affect I_Ca,L_ and I_NCX_
	Involve in tissue fibrosis and structural remodeling
Intracellular Ca^2+^	Ca^2+^ overload and oxidative stress interact, drive fibrosis

#### 3.2.1 Ion channels dysfunction is mediated by mitochondrial oxidative stress injury

Thioredoxin 2 (Trx2) serves as a rate-limiting enzyme in the mitochondrial thioredoxin system, which is one of the principal pathways for scavenging reactive oxygen species (ROS). Trx2 can bind to apoptosis signal-regulated kinase-1 (ASK1), inhibiting its activity and thereby suppressing apoptosis induced by the pro-apoptotic factor cysteine-aspartic acid protease-3 (Caspase3) (146, 147). This mechanism plays a pivotal role in maintaining cellular survival, reducing oxidative stress, and regulating mitochondrial apoptosis signal transduction (148–150). Bicheng Yan et al. discovered that Trx2 is essential for maintaining HCN4-mediated normal heart rate; the absence of Trx2 results in significant ROS accumulation, leading to the dysfunction of HCN4, a critical factor in the development of SSS (151). Carlos H. Pereira et al. found that p21-activated kinase 1 (Pak1) enhances NADPH oxidase 2-dependent ROS production, thereby reducing HCN expression and inhibiting sinoatrial node activity. The sinoatrial node dysfunction, associated with a decreased heart rate following oxidative stress injury controlled by Pak1, is linked to membrane clock dysfunction rather than Ca^2+^ clock dysfunction. Additionally, the application of TEMPOL, a ROS scavenger, can clear ROS and reverse such sinoatrial node function impairment (152). It is evident that HCN4 is an ion channel significantly affected by mitochondrial oxidative stress (152). A large amount of ROS accumulated by oxidative stress damage can affect the function of K_ATP_ (153), the process of tachypacing-induced mitochondrial dysfunction is often accompanied by oxidative stress damage and Ca^2+^ overload, targeted use of antioxidants can reverse associated mitochondrial dysfunction, which reduced ADP and increased ATP production in cells, meanwhile, K_ATP_ expression increased (154). The cardiac sodium channel NaV1.5 (SCN5A) has a fundamental role in excitability and conduction, peroxisome proliferator activated receptor-γ (PPARγ) coactivator-1 (Pgc-1)-deficient murine cardiac models simulate mitochondrial dysfunction, a decrease in Nav1.5 channel protein expression was found in these models (155). Upon tetrodotoxin (TTX) exposure, it is showed that ROS increase, mitochondrial function decreases, SCN5A expression decreased (156). As we can see, mitochondrial oxidative stress injury is an important factor affecting the function and expression of SCN5A, meliorate mitochondrial oxidative stress and preserve bioenergetics can improve mitochondrial dysfunction and protect the function of SCN5A (157). Angiotensin II (Ang II) enhances the expression of KV1.5 (KCNA5) by activating ROS-dependent phosphorylation of Smad2/3 (forming P-Smad2/3) and ERK 1/2 (forming P-ERK1/2), antioxidant can diminish Ang II-induced reactive oxygen species (ROS) generation, inhibit Ang II-induced expression of P-Smad2/3, phosphorylated ERK (P-ERK1/2), which maintains the normal function of KV1.5 (158–160).Trx2 is regulated by nuclear factor-erythroid 2-related factor 2 (Nrf2), which together constitute a crucial component of mitochondrial oxidative stress (161, 162). Heng Zhang et al. found that SSS is associated with oxidative stress damage, which may be related to the loss of HCN4 and the weakening of I_f_ in the process of oxidative stress, and the anti-oxidative stress effect of regulating the Nrf-2/HO-1 axis can improve SSS (163).

#### 3.2.2 Mitochondrial oxidative stress and Ca^2+^/CaMKII activation participate in Ca^2+^ clock and structural remodeling

During mitochondrial oxidative stress, a substantial accumulation of ROS participates in the calcium release process of RyRs on the SR (164). This phenomenon is related to ROS-mediated phosphorylation of CaMKII, where aberrant activation of CaMKII phosphorylation results in Ca^2+^ leakage of RyRs and abnormal LCR (165, 166). LCR affects the discharge rhythm and the recycling and release of cytoplasmic Ca^2+^ by SR, I_Ca,L_ and I_NCX_ (128, 167). Duanyang Xie et al. identified that mitochondrial excitatory amino acid transporter 1 (EAAT1)-dependent mitochondrial glutamate input enhances ROS production, leading to the oxidation of CaMKII protein, ultimately augmenting LCR (168). Jian-Bin Xue et al. observed that sinoatrial node dysfunction following heart failure manifested as decreased CaMKII phosphorylation, reduced RyRs protein expression, diminished SERCA function, lowered SR Ca^2+^ content, attenuated LCR, and inhibited Ca^2+^ clock. Furthermore, CaMKII was implicated in subsequent tissue fibrosis and structural remodeling during this process (169, 170). Despite appearing contradictory, the findings of the two scholars are reconcilable. Duanyang Xie's observations pertain to glutamate-mediated ROS generation within the controlled oxidative stress damage range of sinoatrial node cells, whereby sufficient ROS and substrates required by CaMKII can enhance LCR. Under severe oxidative stress, CaMKII often mediates apoptosis, exacerbating the initial injury (171, 172). Jian-Bin Xue's findings are predicated on sinoatrial node dysfunction post-heart failure, where CaMKII contributes more significantly to tissue fibrosis and structural remodeling as part of compensatory mechanisms. Both accelerated and decelerated heart rates mediated by LCR enhancement or attenuation align with clinical manifestations of SSS, including fast-slow syndrome and slow-fast syndrome. Our inferences, based on clinical presentations, suggest that CaMKII activation mediated by mitochondrial oxidative stress injury and the abnormality of the sinoatrial node Ca^2+^ clock LCR are experimentally demonstrated, also involving fibrosis and remodeling.

#### 3.2.3 Mitochondrial Ca^2+^ overload and oxidative stress damage mediated fibrosis

Mitochondria affect intracellular Ca^2+^ concentration by modulating Ca^2+^ absorption and release. When Ca^2+^ overload occurs in mitochondria, mitochondrial membrane potential changes will cause oxidative stress or aggravate mitochondrial oxidative stress damage. ROS generated by oxidative stress can further damage mitochondrial membrane structure, affecting mitochondrial Ca^2+^ uniporter (MCU) and mitochondrial permeability transition pore (mPTP), thereby aggravatingCa^2+^ overload (173–175). Mitochondrial Ca^2+^ overload and oxidative stress often interact (176). Xing Chang et al. demonstrated that hypoxia/reoxygenation (H/R) injury disrupts the equilibrium of “Ca^2+^ release” and “Ca^2+^ cycling” in rat sinoatrial node cells, and abnormal Ca^2+^ concentration or Ca^2+^ overload in sinoatrial node cells further aggravated mitochondrial oxidative stress injury (177). Oxidative stress and Ca^2+^ overload are frequently implicated in the pathogenesis of fibrosis (178–180). An imbalance in Ca^2+^ homeostasis is a critical factor in sinoatrial node dysfunction and regional tissue fibrosis. Mingjie Zheng et al. found that Hippo-Yap pathway plays a role in maintaining sinoatrial node homeostasis by regulating Ca^2+^ homeostasis, and the inactivation of Lats1/2, pathologically associated with the Hippo-Yap signaling pathway, results in severe dysfunction of the sinoatrial node, which is manifested by Ca^2+^ homeostasis imbalance and increased fibrosis in the sinoatrial node region (181). Impulse conduction disorders in the atrial region are still an important part of SSS, and mitochondrial oxidative stress in atrial myocytes is a significant cause of atrial fibrosis (182).

### 3.3 Mitochondrial quality control (MQC) and SSS mechanism

MQC refers to the mechanisms within the cell that regulate the number, morphology and function of mitochondria to ensure their normal functioning and cell health, and involves mitochondrial protein homeostasis, mitochondrial autophagy, and mitochondrial dynamics and biogenesis (183–185). MQC defects often play a crucial role in the pathogenesis of cardiovascular diseases (186), especially degenerative diseases correlated with aging (187). MQC defects associated with the pathogenesis of SSS have been reported mainly in fusion protein-mediated mitochondria-SR coupling and dynamin-related protein mediated mitochondria fission and autophagy (Table 4).

**Table 4:** Summary of MQC and SSS mechanism.

Action target	Mechanism of action	
Mfn2	Normal expression	Maintain inter-organelle contact and communication between mitochondria and SR, involve in LCR
	Decreased expression	Increase mitochondria-SR distance, and affect LCR
Drp1	Normal expression	Synergize with the PINK1/Parkin signaling axis to mediate mitochondrial autophagy, ensure the stable level of MCQ
	Overactivation	Induce apoptosis, inhibit Drp1 can prevent excessive mitochondrial fission, activates mitophagy, improve SSS

#### 3.3.1 Mitofusin 2(Mfn2) mediated mitochondria-SR coupling

Mfn2 is a transmembrane guanosine triphosphatase (GTPase) located in the outer membrane of mitochondria, and a key factor in regulating mitochondrial fusion and maintaining mitochondrial structure (188). Mfn2 is also expressed on SR, and the proximity between mitochondria and SR (189), facilitates a hub for crosstalk between them (190, 191). Inter-organelle contact and communication between mitochondria and SR maintain cellular homeostasis (192), especially in the Ca^2+^ cycle involved in LCR (189, 190, 193, 194). Under normal conditions, mitochondrial ATP is vital to power SR Ca^2+^ cycling that drives phasic contraction/relaxation, and changes in SR Ca^2+^ release are sensed by mitochondria and used to modulate oxidative phosphorylation based on metabolic need (195). Disruption of the physical link between SR and mitochondria mediated by Mfn2 impairs the mitochondria-SR metabolic feedback mechanism. This results in diminished SERCA activity, obstructed Ca^2+^ cycling, impaired mitochondrial ATP production, disrupted energy metabolism, and potentially programmed cell death (195, 196). Lu Ren et al. identified that sinoatrial node dysfunction is associated with Mfn2-mediated alterations in mitochondria-SR connectomics. In a mouse model of sinoatrial node dysfunction induced by heart failure, electron microscopy (EM) tomography revealed mitochondrial structural abnormalities and increased mitochondria-SR distance. This results in abnormal mitochondrial Ca^2+^ processing, altered local PKA activity, and impaired mitochondrial function in sinoatrial node cells (197).

#### 3.3.2 Dynamin-related protein 1 (Drp1) mediates mitochondrial fission and autophagy

Drp1, a GTPase widely distributed in the cytoplasm, is a major regulatory factor in the process of mitochondrial fission (198, 199). When the mitochondrial fission process is activated, Drp1 is transported to the mitochondrial surface, where it binds to related receptors to form helical oligomers. These helical Drp1 structures facilitate its GTP hydrolysis (200, 201), encircle the mitochondrial outer membrane, and mediate its scission (202, 203). Drp1 plays a major role in the entire process of mitochondrial fission (204), DRP1-mediated mitochondrial fission promotes the occurrence of mitochondrial autophagy, and the dysfunctional mitochondria produced by fission depend on the clearance of mitochondrial autophagy (205, 206). Under normal cellular conditions, Drp1 synergizes with the PINK1/Parkin signaling axis to mediate mitochondrial autophagy, ensuring the stable mitochondrial quality level and function (207). Excessive activation of Drp1 leads to excessive mitochondrial fission beyond the scope of mitochondrial autophagy, the increase of Drp1 level promotes excessive opening of mPTP, oligomerization of BCL2-associated X protein (Bax) and release of Cyt C, and mitochondrial ROS accumulation, and loss of mitochondrial membrane potential, ultimately inducing apoptosis (208, 209). Conversely, the application of Drp1 inhibitors can ameliorate these adverse effects (210, 211). Rebecca Z Fan et al. found that a partial Drp1 knockout improves autophagy (212). Mitochondrial fission is integral to fibrosis, as evidenced by Ching-Yi Chen et al., who found that Drp1 inhibition elevated ATP levels and reduced mitochondrial fission and apoptosis, thereby mitigating fibrosis (213). Enhanced mitochondrial autophagy also contributed to the reduction of ECM in the sinoatrial node (33), the mechanism of fibrosis in SSS is linked to Drp1-mediated mitochondrial fission and autophagy. Xing Chang et al. proved that Tongyang Huoxue decoction (TYHX) inhibits Drp1 translocation to mitochondria, prevents excessive mitochondrial fission, activates mitophagy, and enhances mitochondrial membrane potential, demonstrating TYHX's efficacy in improving SSS (177).

**Figure 1:**
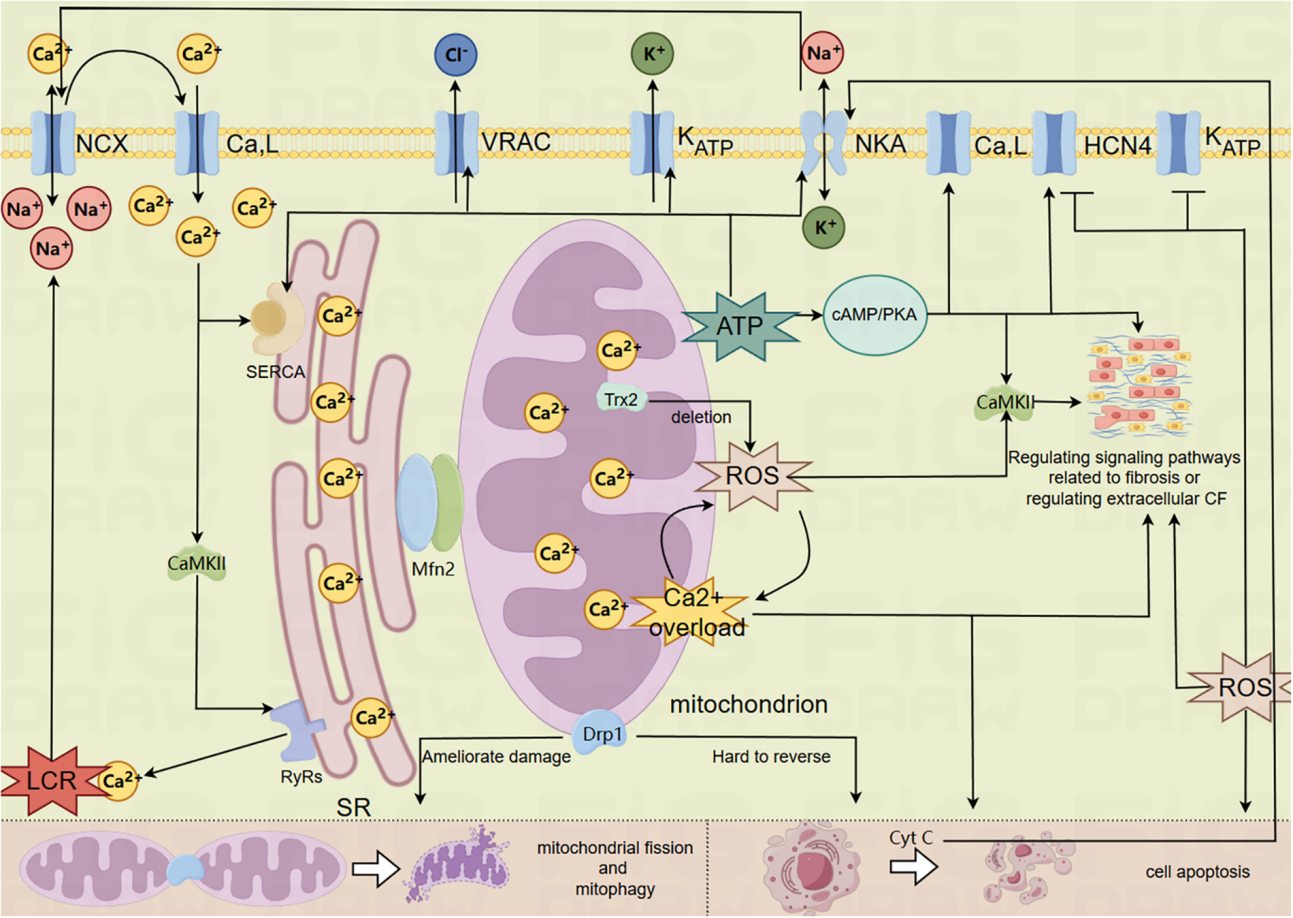
Mechanism of mitochondrial involvement in SSS. (The model membrane clock ion channel proteins in the figure are closely related to mitochondria and have been mentioned in relevant literature, so they cannot represent all membrane clock ion channel proteins in the figure).

The foregoing is the process of mitochondria related mechanisms participating in the pathological mechanism of SSS, which is mainly reflected in mitochondrial energy metabolism, mitochondrial oxidative stress damage, MCQ's involvement in the basic pathological mechanism of SSS regarding the exacerbation of regional tissue fibrosis and the dysfunction of the coupled-clock system mechanism. For the summary, please refer to the mechanism diagram (Figure 1).

## 4 Summary and conclusion

The rhythmically beating heart is an organ with high energy demand (214). Mitochondrial dysfunction mechanisms are frequently implicated in the physiological and pathological processes of various cardiovascular diseases, including myocardial ischemia-reperfusion injury (215), atrial fibrillation (216), heart failure (217) and recovery after myocardial infarction (218). There is no doubt that SSS cannot be excluded, particularly as this class of diseases is clearly associated with aging, which has been shown to correlate with mitochondrial dysfunction (219, 220). Disruption of the coupled-clock system mechanism and severe regional tissue fibrosis are the fundamental mechanisms of SSS. ATP and its derivative, cAMP, produced via mitochondrial energy metabolism, are involved in regulating membrane clock-related ion channel proteins and Ca^2+^ clock-related ion pumps, and in modulating signaling pathways that mediate fibrosis or extracellular CF. Additionally, mitochondria-mediated oxidative stress damage, Ca^2+^ overload, and MCU dysfunction contribute to the development and progression of these mechanisms in SSS.

In fact, SSS is a kind of syndrome of pacing function and (or) impulse conduction dysfunction caused by dysfunction of sinoatrial node and surrounding tissue lesions. This syndrome encompasses lesions in the sinoatrial node region as well as in the adjacent atrial and atrioventricular junction areas, complicating both clinical and experimental research efforts. Clinical diagnosis primarily relies on electrocardiographic evaluations, yet the diverse and complex electrocardiographic manifestations across different heart regions further complicate the classification of disease types. At the same time, various animal modeling methods of SSS often fail to reflect the complexity of clinical diseases, such as gene regulation (23), inducing ischemia-reperfusion injury on the sinoatrial node area, and injecting sodium hydroxide to the sinoatrial node area through internal jugular vein (221), among others. On the other hand, mitochondria are ingenious and magical cellular organelles with powerful functions. They can regulate energy metabolism, oxidative stress and Ca^2+^ level, communicate with other neighboring organelles, participate in intracellular communication between organelles and mitochondrial autophagy to clear dysfunctional mitochondria and maintain intracellular environmental homeostasis. At the same time, mitochondrial dysfunction to the point of inability to stabilize the entire cell function can mediate the occurrence of apoptosis. The multifaceted roles of mitochondria are interdependent; for instance, oxidative stress damage can be exacerbated by abnormal energy metabolism or Ca^2+^ overload in mitochondria. On the contrary, energy metabolism is influenced by mitochondrial oxidative stress and Ca^2+^ concentrations, with oxidative stress and Ca^2+^ overload mutually interacting. Mitochondrial dynamics, such as fission, fusion, and autophagy, are integral to maintaining complete mitochondrial functionality, thus adding to the complexity and challenges of related studies.

Based on the published clinical and experimental findings concerning the mechanisms of mitochondrial dysfunction and SSS, it is evident that further investigation is warranted. Specifically, it remains unclear whether other ion channel proteins associated with the membrane clock are directly regulated by mitochondrial mechanisms and contribute to the fundamental pathology of SSS, or if they exert no significant regulatory effects. Additionally, the role of mitochondria in the fibrosis process mediated by certain ion channel proteins, which may be central to the basic mechanism of SSS, requires further elucidation. Therefore, current research in this area is insufficient. Further in-depth and comprehensive mechanism studies will help clarify the involvement of mitochondrial mechanism in the pathological mechanism of SSS and identify the core link of disease pathogenesis, which is conducive to the development of relevant new effective drugs to prevent and treat SSS.
